# 

**Published:** 1886-11-20

**Authors:** 


### TABLE OF CONTENTS.

### Original and Selected Articles.

Puerperal Eclampsia, by Walter A. Crow, M.D., Lecturer on Diagnosis in the Southern Medical College, Atlanta, Ga .......... 406
Case of Multiple Abscess, by J McF. Gaston, M.D., Professor o the Principles and Practice of Surgery .......... 414
Properties and Dosage of Chloranodyne .......... 416
How to Administer Quinine in Malarial Intermittent Fevers .......... 418
Differentiation Between Diphtheria and Membranous Croup, by Dr. W. A. Morris, M D., Austin, Texas .......... 419
Nitrate of Silver in Whitlow .......... 420
An Unusual Case of Post-Partum Hemorrhage, by P. C. Williams, M. D .......... 421

### Abstracts and Gleanings.

The Excretion of Drugs by the Mammary Glands. .......... 425
Another Application for Antipyrine .......... 426
Influence of Mental Impressions on the Fœtus; Medical Men as Witnesses and Experts .......... 427
Dysentery .......... 428
Treatment of Acute Rheumatism; The Treatment of Gleet .......... 429
Ptomaines .......... 430
Egg Diet; Diphtheria .......... 431
An Anodyne for Use in Vesical Irritation; The Treatment of Ulcerative Otorrhœa .......... 432
Ten Commandments for Bathers; Treatment for Diphtheria; Reflex Pain. .......... 433
Turpentine as an Antidote to Phosphorus; Squills in Whooping-Cough; Piperine .......... 434

### Scientific Items.

Development of the Human Body .......... 335
Comparative Longevity of the Sexes .......... 436

### Practical Notes and Formulæ.

Treatment of Diphtheria; Speedy Cure of Gonorrhœa; Lemonade Iron. .......... 437

Anise Cordial or Fennel Gin; Apone: A New Kind of Pain Expeller; Catarrhal Headache; How they do it in China. .......... 438

### Editorials and Miscellaneous.

Editorial Briefs .......... 439

Prison Reform; The Anatomical Bill .......... 440

Books and Pamphlets Received. .......... 442

Receipted; Special Notices .......... 444

